# Paraspeckle components NONO and PSPC1 are not mislocalized from motor neuron nuclei in sporadic ALS

**DOI:** 10.1093/brain/awaa205

**Published:** 2020-08-10

**Authors:** Giulia E Tyzack, Giulia Manferrari, Jia Newcombe, Nicholas M Luscombe, Raphaelle Luisier, Rickie Patani

**Affiliations:** a1 The Francis Crick Institute, London NW1 1AT, UK; a2 Department of Neuromuscular Diseases, UCL Queen Square Institute of Neurology, Queen Square, London, UK; a3 NeuroResource, Department of Neuroinflammation, UCL Queen Square Institute of Neurology, London, UK; a4 UCL Genetics Institute, University College London, London WC1E 6BT, UK; a5 Okinawa Institute of Science and Technology Graduate University, Okinawa 904-0495, Japan; a6 Idiap Research Institute, Centre du Parc, Office 206, PO Box 592, CH-1920 Martigny, Switzerland

##  

Amyotrophic lateral sclerosis (ALS) is a fatal neurodegenerative disease characterized by the progressive loss of upper and lower motor neurons. Although its precise aetiopathogenesis remains unclear, cellular hallmarks of the disease include the deregulation of RNA metabolism and the mislocalization of RNA binding proteins (RBPs) from the nucleus to the cytoplasm (Butti and Patten, 2018). We recently showed that FUS exhibits nuclear loss in sporadic ALS (Tyzack *et al*., 2019). This finding builds on our earlier discovery of nuclear loss of SFPQ in ALS (Luisier *et al*., 2018) and the established hallmark of TAR DNA-binding protein 43 (TDP-43, encoded by *TARDBP*) nuclear-to-cytoplasmic mislocalization (Neumann *et al*., 2006). These three proteins are components of paraspeckles, nuclear ribonucleoprotein membraneless compartments (or granules) formed in the interchromatin space together with the long non-coding RNA (lncRNA) *NEAT1_2* (Clemson *et al*., 2009; Sasaki *et al*., 2009; Mao *et al*., 2011).

Paraspeckles are involved in the regulation of normal gene expression via sequestration of transcription factors and RBPs, in the nuclear retention of some specific classes of transcripts, and in pri-miRNA processing (Nakagawa *et al*., 2018). While in healthy motor neurons paraspeckles are absent due to the lack of expression of *NEAT1_2*, enhanced paraspeckle formation has been observed in both sporadic (Nishimoto *et al*., 2013; Shelkovnikova *et al*., 2018) and familial ALS cases (Shelkovnikova *et al*., 2018; An *et al*., 2019), and is proposed to represent a protective neuronal response to stress.

In paraspeckles, RBPs are arranged with a core and shell structure (Fig. 1C). RBPs in the core are fundamental for the structural integrity of paraspeckles and include FUS and the DBHS family members SFPQ, NONO and PSPC1, while TDP-43 is found in the shell (West *et al*., 2016). Proteins in this family share a similar protein domain architecture and evolutionary origin, and they are known to form homodimers or heterodimers with other DBHS proteins (Knott *et al*., 2016). Noting that at least three paraspeckle components are lost from the nuclei of ALS motor neurons (Neumann *et al*., 2006; Luisier *et al*., 2018; Tyzack *et al*., 2019), we sought to address the hypothesis that nuclear dislocation is a more widespread phenomenon affecting other paraspeckle proteins, specifically NONO and PSPC1.

**Figure 1:**
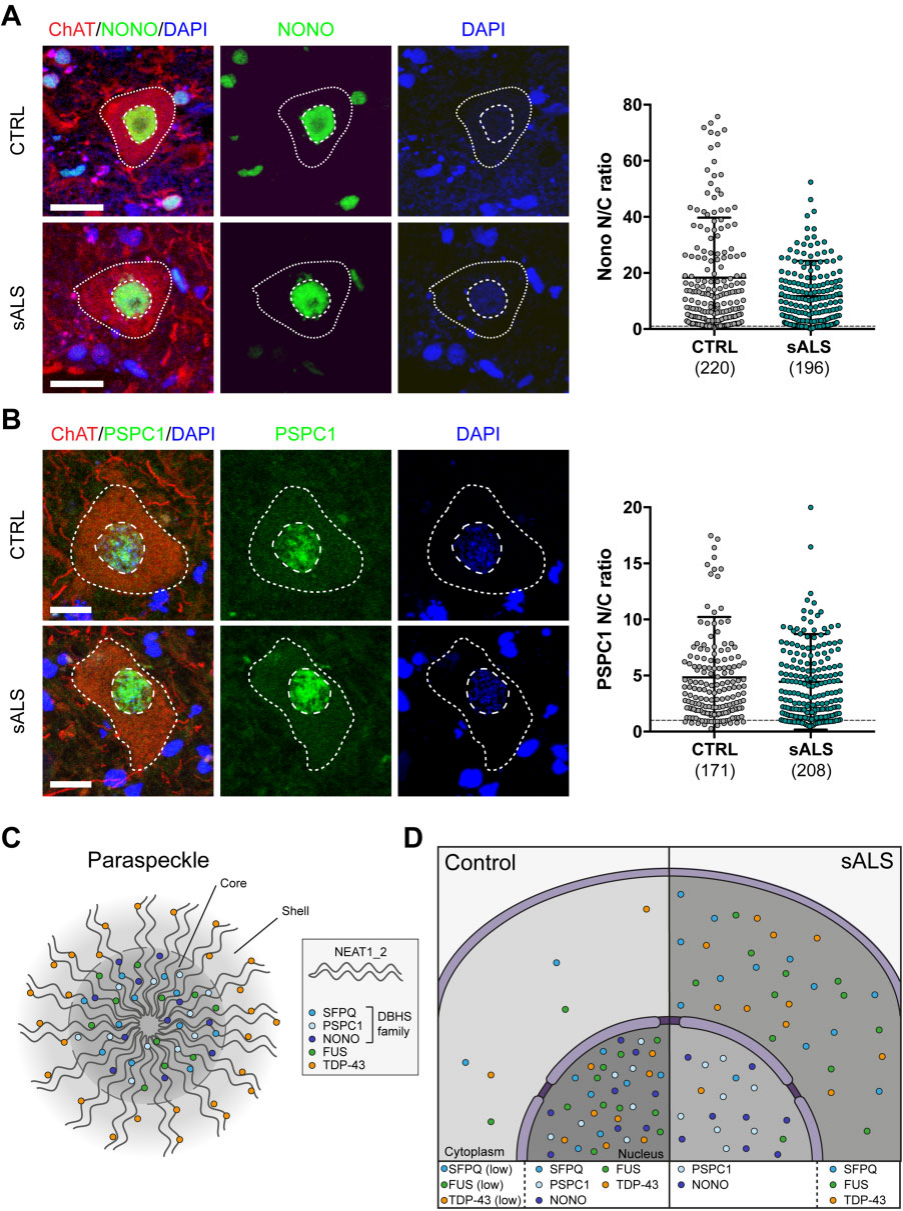
**NONO and PSPC1 are not lost from motor neuron nuclei in sporadic ALS.** (**A** and **B**) Analysis of the subcellular localization of NONO (**A**) and PSPC1 (**B**) in motor neurons in the ventral spinal cord of healthy control subjects (CTRL, *n *=* *8) and patients with sporadic ALS (sALS, *n *=* *12) from the same cohort analysed in our previous study (Tyzack *et al*., 2019). Motor neuron cytoplasm was identified by ChAT immunohistochemistry (red), nuclei were counterstained with DAPI (blue). NONO and PSPC1 are shown in green in **A** and **B**, respectively. Images were acquired as confocal *z*-stacks using a Zeiss 710 confocal microscope with a *z* step of 1 μm, processed to obtain a maximum intensity projection and analysed using Fiji. The nuclear and cytoplasmic areas were manually drawn based on DAPI and ChAT staining, respectively. For each region of interest, the average immunoreactivity intensity for each RBP was measured, the background was subtracted, and the ratio between nuclear and cytoplasmic average intensity was calculated. Data shown in the dot plots are nuclear/cytoplasmic (N/C) ratio (mean ± SD) per cell, with the dashed line set at a N/C ratio of 1. The total number of cells analysed is shown for each group. Scale bar = 20 μm. Linear mixed effect analysis was used to test the relationship between NONO or PSPC1 localization and sporadic ALS, thus accounting for individual case-based idiosyncratic variation. *P*-values were obtained by likelihood ratio tests of the full model with the effect in question against the model without the effect in question: *P*-value (NONO) = 0.37 and *P*-value (PSPC1) = 0.78. (**C**) Paraspeckle structure showing the localization of different RBPs in the core or shell. (**D**) Schema summarizing the findings of N/C distribution of different paraspeckle components in human sporadic ALS.

Here, we examined the nuclear/cytosolic ratio of NONO and PSPC1 in lumbar spinal cord motor neurons of eight sporadic ALS cases and 12 age- and gender-matched controls using immunohistochemistry, as previously described (Tyzack *et al*., 2019). In contrast to the reported nuclear displacement of FUS and SFPQ, we observed comparable nuclear/cytosolic distributions of both NONO and PSPC1 in all samples (NONO: control = 18.35 ± 21.38, sporadic ALS = 11.79 ± 12.5; PSPC1: control = 4.84 ± 5.39, sporadic ALS = 4.44 ± 4.25) (Fig. 1A and B). Taken together with aforementioned studies, these data suggest that some paraspeckle components (TDP-43, SFPQ and FUS), but not others (NONO and PSPC1) exhibit nuclear loss in sporadic ALS (Fig. 1C and D).

To our knowledge this is the first study investigating the subcellular localization of PSPC1 in ALS-affected motor neurons. Previous work by Shelkovnikova *et al*. (2014) described NONO co-localization with cytoplasmic FUS aggregates in mouse models and ALS patients carrying FUS mutations. However, in line with our findings, this was not detected in the cytosol of sporadic cases, or SOD1-ALS patients, suggesting that sequestration of NONO to cytoplasmic aggregates represents a pathological feature that is specific to FUS mutations (Shelkovnikova *et al*., 2014). The fact that, while sharing structure and function with the other DBHS proteins, SFPQ is the only RBP of this family that is lost from the nucleus of ALS motor neurons is somewhat surprising. Despite substantial overlap, there are also some remarkable differences in the role of DBHS proteins in regulating gene expression, splicing, transcript localization and stability (Knott *et al*., 2016). In specific circumstances, and in a cell type-specific manner, this is also reflected in a differential localization of these proteins. For example, PSPC1, but not SFPQ nor NONO, is localized to the cytoplasm during adipocyte differentiation (Wang *et al*., 2017). In neurons, in addition to its nuclear localization, SFPQ is also found in axons, where it is required for correct axon development and maintenance of axon viability through the local transport of specific classes of transcripts (Cosker *et al*., 2016; Thomas-Jinu *et al*., 2017). The ability of SFPQ to shuttle between the nucleus and the cytoplasm, and its physiological cytoplasmic roles in mature neurons, might, at least in part, explain its susceptibility to nuclear loss under pathological conditions.

Lastly, our observation that NONO and PSPC1 retain a predominantly nuclear localization in ALS does not necessarily exclude a loss of their nuclear physiological functions. Their sequestration within subnuclear bodies is a possibility that remains open, and yet to be fully addressed. For example, paraspeckle components, including SFPQ, FUS, NONO, and PSPC1 are sequestered by G4C2 nuclear foci in C9ORF72 patient-derived fibroblasts (Bajc Česnik *et al*., 2019). Similarly, in light of the obligatory dimerization of DBHS proteins, the nuclear loss of SFPQ in ALS might alter the balance and composition of these dimers, potentially affecting the function of nuclear NONO and PSPC1.

### Data availability

Data supporting the findings of this study are available from the corresponding authors, upon reasonable request.

## Funding

This work was supported by the Francis Crick Institute which receives its core funding from Cancer Research UK (FC010110), the UK Medical Research Council (FC010110), and the Wellcome Trust (FC010110). R.P. holds an MRC/MND Association Lady Edith Wolfson Senior Clinical Fellowship [MR/S006591/1] and is supported by the National Institute for Health Research University College London Hospitals Biomedical Research Centre. N.M.L. is supported by a Wellcome Trust Senior Investigator Award [103760/Z/14/Z], an MRC eMedLab Medical Bioinformatics Infrastructure Award to N.M.L. (MR/L016311/1). N.M.L. is a Winton Group Leader in recognition of the Winton Charitable Foundation’s support towards the establishment of the Francis Crick Institute. R.L. is supported by the Idiap Research Institute.

## Competing interests

The authors report no competing interests.
